# On the Cerebral Convolutions of Man

**Published:** 1874-02

**Authors:** 


*On The Cerebral Convolutions of Man, Represented according to Observations, especially upon their Development in the Fœtus.* By Alexander Ecker, Prof. etc. Translated by Robert T. Edes, M. D. New York: D. Appleton & Co., 1873. Buffalo: Martin Taylor.

Dr. Ecker has in this monograph given the results of a vast amount of laborious research and study. It has been his object to place in the hands of physicians, a guide whereby they may be directed in their study of the Human Cerebral Convolutions. This is a work which has been attempted in a measure by others, but in most instances with but little satisfaction; how well the present writer has succeeded, careful study can only disclose. From a hurried reading we are inclined to think that he has made a valuable addition to the works on the anatomy of the brain.

